# Multiparametric exercise stress cardiovascular magnetic resonance in the diagnosis of coronary artery disease: the EMPIRE trial

**DOI:** 10.1186/s12968-021-00705-8

**Published:** 2021-03-04

**Authors:** Thu-Thao Le, Briana W. Y. Ang, Jennifer A. Bryant, Chee Yang Chin, Khung Keong Yeo, Philip E. H. Wong, Kay Woon Ho, Jack W. C. Tan, Phong Teck Lee, Calvin W. L. Chin, Stuart A. Cook

**Affiliations:** 1 National Heart Research Institute Singapore, National Heart Centre Singapore, 5 Hospital Drive, Singapore, 169609 Singapore; 2 Cardiovascular Sciences ACP, Duke-NUS Graduate Medical School, Singapore, Singapore; 3 Department of Cardiology, National Heart Centre Singapore, Singapore, Singapore; 4 National Heart and Lung Institute, Imperial College, London, UK

**Keywords:** Exercise stress, Supine cycle ergometer, Coronary artery disease, Cardiovascular magnetic resonance, Fractional flow reserve

## Abstract

**Background:**

Stress cardiovascular magnetic resonance (CMR) offers assessment of ventricular function, myocardial perfusion and viability in a single examination to detect coronary artery disease (CAD).

We developed an in-scanner exercise stress CMR (ExCMR) protocol using supine cycle ergometer and aimed to examine the diagnostic value of a multiparametric approach in patients with suspected CAD, compared with invasive fractional flow reserve (FFR) as the reference gold standard.

**Methods:**

In this single-centre prospective study, patients who had symptoms of angina and at least one cardiovascular disease risk factor underwent both ExCMR and invasive angiography with FFR. Rest-based left ventricular function (ejection fraction, regional wall motion abnormalities), tissue characteristics and exercise stress-derived (perfusion defects, inducible regional wall motion abnormalities and peak exercise cardiac index percentile-rank) CMR parameters were evaluated in the study.

**Results:**

In the 60 recruited patients with intermediate CAD risk, 50% had haemodynamically significant CAD based on FFR. Of all the CMR parameters assessed, the late gadolinium enhancement, stress-inducible regional wall motion abnormalities, perfusion defects and peak exercise cardiac index percentile-rank were independently associated with FFR-positive CAD. Indeed, this multiparametric approach offered the highest incremental diagnostic value compared to a clinical risk model (*χ*^*2*^ for the diagnosis of FFR-positive increased from 7.6 to 55.9; *P* < 0.001) and excellent performance [c-statistic area under the curve 0.97 (95% CI: 0.94–1.00)] in discriminating between FFR-normal and FFR-positive patients.

**Conclusion:**

The study demonstrates the clinical potential of using in-scanner multiparametric ExCMR to accurately diagnose CAD.

*Trial registration:* ClinicalTrials.gov, NCT03217227, Registered 11 July 2017–Retrospectively registered, https://clinicaltrials.gov/ct2/show/NCT03217227?id=NCT03217227&draw=2&rank=1&load=cart

## Introduction

Stress cardiovascular magnetic resonance (CMR) has evolved as a highly accurate non-invasive diagnostic [1–3] and prognostic [4–6] test that provides cost-effective imaging-based strategy to guide coronary revascularization [7, 8] in patients with stable coronary artery disease (CAD). Advantages of CMR include its excellent spatial resolution, independence of acoustic windows, free from ionising radiation and multi-parametric assessment of ventricular function, myocardial perfusion and viability in a single examination.

Exercise stress tests are more physiological in replicating the symptoms and haemodynamic changes compared to pharmacological-induced stress, with less adverse events and better tolerated [9–11]. However, due to limited availability of CMR-conditional stress equipment that precludes physiological-induced stress, conventional stress CMR typically refers to pharmacological-induced stress perfusion CMR [12]. Only recently has exercise treadmill CMR made inroads into the diagnosis of CAD, with a multi-centre trial demonstrated excellent agreement between exercise treadmill CMR and coronary angiography [13].

We have developed an exercise stress CMR (ExCMR) protocol using an in-scanner cycle ergometer [14] and established normal exercise capacities (peak exercise cardiac index percentile-rank; Peak_CI_) in the local healthy population [15]. The in-scanner stress protocol allows imaging to be done at every stage of exercise. Peak_CI_ has been validated against the maximal oxygen uptake (VO_2_max) of cardiopulmonary stress test [14] and a low Peak_CI_ (< 35th percentile for age and sex) discriminates between pathological and physiological cardiac remodelling [15]. Extending this work, we aim to examine the diagnostic value of ExCMR in patients with suspected CAD, compared with the gold standard invasive fractional flow reserve (FFR) to define significant CAD. We hypothesized that a multiparametric approach of CMR parameters would have a high diagnostic accuracy for CAD.

## Methods

### Study design and patient population

The EMPIRE trial (Evaluating Myocardial Ischaemia in Chest Pain Using Exercise CMR; ClinicalTrials.gov identifier: NCT03217227) is a single-centre prospective study. The patients who had symptoms of angina (Canadian Class Symptoms CCS class II or III [16]) and at least one cardiovascular disease risk factor (diabetes, hypertension, dyslipidaemia, smoking, or family history of CAD). Those patients who were recommended for invasive coronary angiograms by their cardiologists and agreed to participate in the study were recruited. Exclusion criteria were previous coronary artery interventions (coronary artery bypass grafting; and/or percutaneous coronary intervention), acute coronary syndromes (unstable angina, non-ST elevation myocardial infarction and ST elevation myocardial infarction), physical disabilities that would preclude exercise testing, contraindications to contrast-enhanced CMR, and inability to tolerate adenosine (for FFR assessment).

The study was conducted in accordance to the Declaration of Helsinki and approved by the Singhealth Centralised Institutional Review Board. Written informed consent was obtained from all participants prior to CMR and invasive coronary angiogram.

### Exercise CMR protocol

Patients taking beta blockers were advised to stop taking them 2 days before stress test. Baseline breath-hold CMR cine images and native T1 maps were acquired in all patients before initiating exercise stress (60-channel phased-array coil, MAGNETOM Aera 1.5T, Siemens Healthineers, Erlangen, Germany). ExCMR was performed with a programmable supine ergometer (Lode BV, Groningen, the Netherlands) fitted onto the CMR scanner table. Patients were asked to cycle at the initial workload of 25 W, with cadence maintained at least 70 rpm for 1 min. Workload was increased by 25 W every minute until exhaustion or presence of symptoms (dyspnoea or chest pain). Free-breathing imaging was done at the end of every stage during a brief period of stopping exercise to minimize motion and electrocardiogram (ECG) triggering related artefacts. After free-breathing image acquisition at peak exercise, patients were asked to continue cycling for another minute to maintain the maximal heart rate. On exercise termination, free-breathing first-pass stress perfusion was performed with the injection of 0.075 mmol/kg gadolinium contrast agent (Gadovist; Bayer Pharma AG, Berline, Germany). Rest perfusion imaging was acquired 10 min after stress, with a further injection of 0.075 mmol/kg gadolinium, at the same slice locations. Late gadolinium enhancement (LGE) imaging was performed 10 min after the 2nd dose of contrast injection, followed by post-contrast T1 maps at 15 min after contrast. The higher gadolinium dosage of 0.075 mmol/kg was given to maintain the contrast-to-noise ratio for both perfusion and LGE imaging. The total duration of the ExCMR protocol was 60 min. Blood pressure was monitored at every stage of exercise, during stress and rest perfusion imaging and recovery. A five-point score was used to assess patient’s experience at the end of ExCMR:1 = would not do it again, 2 = somewhat uncomfortable, 3 = neutral, 4 = somewhat comfortable, 5 = highly satisfied.

**Fig. 1:**
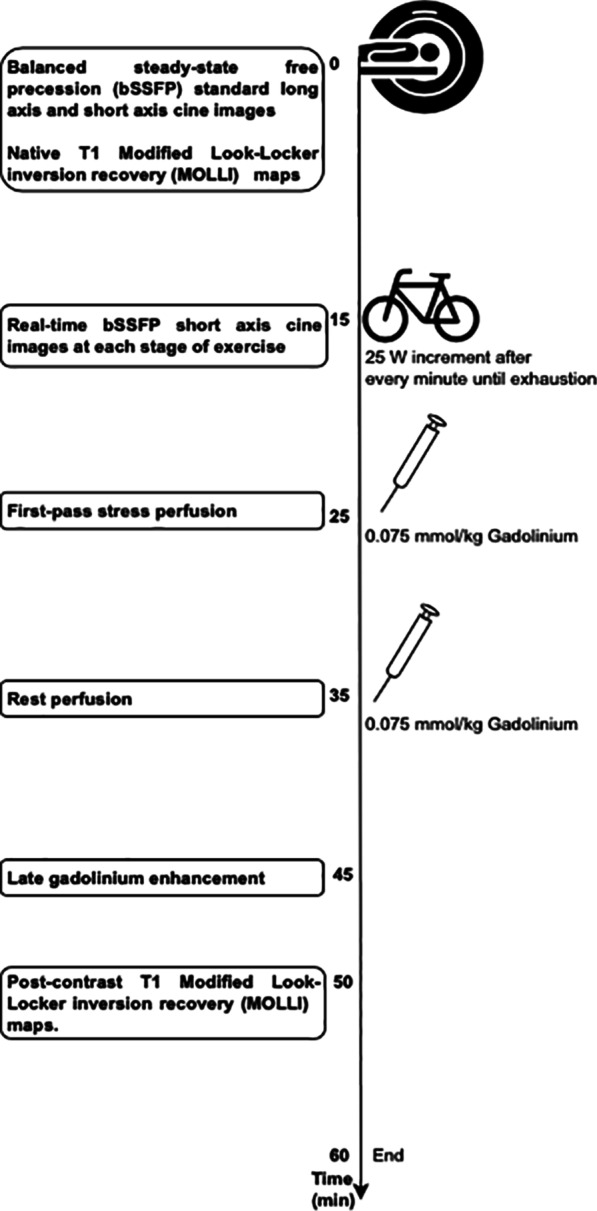
Exercise CMR Protocol and Imaging Parameters

The imaging sequences and parameters were as follows and illustrated in Fig. 1:

#### Breath-hold cines

Balanced steady-state free precession (bSSFP) of long axis (2-, 4-chamber and sagittal left ventricular (LV) outflow tract) and short axis cines extending from base to apex (8 mm thick and 2 mm gap; TE = 1.2 ms; TR = 3 ms; FOV 280–320 mm; 13 segments per phase; acquired matrix size 205 × 256 pixels; acceleration factor 2; acquired voxel size 1.6 × 1.3 × 8 mm; temporal resolution 39 ms; 30 phases per cardiac cycle).

#### Real-time free-breathing cines

bSSFP of short axis cines: prospective ECG-gated free-breathing image acquisition of 10 – 13 short axis slices for at least 2 cardiac cycles, extending from base to apex, at every stage of exercise (8 mm thick and 2 mm gap; TE = 0.99 ms; TR = 2.3 ms; flip angle 56°; FOV 230 × 300 mm; acquired matrix size 68 × 128 pixels; acceleration factor 4; acquired voxel size 3.4 × 2.3 × 8 mm; temporal resolution 39 ms; 10 to 30 phases per cycle depending on heart rate).

#### Stress and rest perfusion

Free-breathing bSSFP sequence of 4 short axis slices over 2 concatenations (slice thickness 8 mm; TE = 0.98 ms; TR = 2.3 ms; flip angle 50°; FOV 263 × 350 mm; acquired matrix size 98 × 160 pixels; acceleration factor 2; acquired voxel size 2.6 × 2.1 × 8 mm). Images were motion corrected by the unsupervised inline process implemented within the Image Calculation Environment of Siemens MR system [17] (Additional file 1).

#### Late-gadolinium enhancement

Phase-sensitive inversion recovery (PSIR) magnitude-reconstructed images of 3 standard long-axis and short-axis stack from base to apex (8 mm thick and 2 mm gap; TE = 1.19 ms; FOV 270 × 360 mm; acquired matrix size 144 × 256 pixels; acceleration factor 2; acquired voxel size 1.9 × 1.4 × 8 mm; inversion time adjusted individually to achieve appropriate nulling of the myocardium).

#### Myocardial native and post-contrast T1 maps

Modified Look-Locker inversion recovery (MOLLI) sequence with 5(3)3 scheme at basal and mid-ventricular levels (8 mm thick; TE = 1.12 ms; FOV 270 × 360 mm; acquired matrix size 144 × 256 pixels; acceleration factor 2; flip angle 35°; acquired voxel size 1.9 × 1.4 × 8 mm; minimum TI = 100 ms and 80 ms increment; inline motion correction).

### CMR image analysis

LV mass and cardiac volumes at baseline (breath-hold short axis cine images) and each exercise stage (real-time short axis cine images) were measured in all patients using standardized protocols at our **NHRIS CMR Core Laboratory** (cvi42, Circle Cardiovascular Imaging, Calgary, Alberta, Canada) [14]. Exercise-related CMR measures assessed in this study included relative change in exercise LV ejection fraction (LVEF), relative change in exercise indexed stroke volume (SVI), relative change in exercise cardiac index, and Peak_CI_ expressed as age- and sex-specific percentiles according to the reference ranges previously established [15]. LGE patterns (ischaemic versus non-ischaemic) and the extent of infarction were visually assessed based on the 16-segment model. Ischaemic LGE pattern was defined as enhancement in a vascular distribution (subendocardial to transmural) [18]. LGE > 50% of myocardial wall thickness was considered non-viable [19]. Native T1 was derived by manually contouring the endocardium and epicardium of the native T1 maps at basal and mid-ventricular levels using standardized protocol [20], excluding infarcted regions as recommended [21]. Ischaemia on T1 maps are regions corresponding to reversible perfusion defects and confirmed by angiographic evidence of significant stenosis [22]. Extracellular volume (ECV) was calculated from native and post-contrast T1 values as:${\text{(1 - haematocrit)}} \times (\Delta {\text{R}}1_{{{\text{myocardium}}}} - \Delta {\text{R}}1_{{{\text{blood}}}})$, where ${\text{R1 = 1/T1}}$. Haematocrit was taken on the day of CMR scan.

Qualitative assessment of inducible regional wall motion abnormalities, perfusion defects and LGE was performed according to contemporary recommendations [23], by two independent CMR physicians prior to invasive coronary angiography. Any discrepancies were resolved by consensus before the coronary angiography was performed. The qualitative components of the ExCMR was recorded as: presence/absence of inducible regional wall motion abnormalities (RWMA), presence/absence of perfusion defects. Presence of hypoperfusion at both stress and rest without corresponding hyperenhancement region on LGE images was considered as artefact. In addition, 9 out of 60 cases (15%) were evaluated by 3 experienced CMR readers to assess the inter-observer reproducibility. All readers were blinded to CMR and FFR reports.

### Invasive coronary angiography and assessment of fractional flow reserve

All patients underwent invasive coronary angiography within 1 month after ExCMR by interventionalists who were briefed to follow the angiography/FFR study protocol. FFR was assessed using standard procedure [24] (PressureWire™ X Guidewire, Abbott Laboratories, Chicago, Illinois, USA), in all coronary arteries with a calibre of 2.5 mm or more and a stenosis severity of 40% or more if technically feasible, as determined by the interventionalist. Maximal hyperaemia was induced by intravenous adenosine infusion at a rate of 180 μg/kg/min for at least 2 min for FFR calculation. An FFR value of ≤ 0.8 or total occlusion was defined as FFR-positive [25]. Total occluded coronary artery was assigned an FFR value of 0.5. The interventionalists were blinded to the findings of the CMR scans before the procedure.

### Statistical analysis

Distribution of continuous variables was assessed using the Shapiro–Wilk test. Data were presented in either mean ± standard deviation or median [inter-quartile range], as appropriate. Depending on the normality of the distribution, parametric Student’s t test and 1-way ANOVA or the nonparametric Mann–Whitney U test and Kruskal–Wallis test were used to compare groups of continuous data. Categorical data were compared using the χ^2^ test. A two-sided P-value < 0.05 was considered as statistically significant. Cohen’s kappa (κ) was used to assess agreement between pairs of readers. The agreement grading based on κ values were: poor (0–0.02), fair (0.21–0.40), moderate (0.41–0.60), substantial (0.61–0.80) and nearly perfect (0.81–1.00).

Determinants associated with FFR-positive were assessed using univariate and backward selection multivariable Firth logistic regression to handle 0 cell frequency. Clinically relevant variables that demonstrated independent association (*P* < 0.10) with significant CAD defined by FFR were retained in the final model [26]. The incremental diagnostic value of the multiparametric model, consisting of CMR variables that were retained from the multivariable analysis, over a clinical model consisting of age, sex and coronary risk factors (diabetes, hypertension, dyslipidaemia and smoking history) and conventionally used CMR parameters for assessment of CAD (RWMA, perfusion defects and LGE) was assessed using a change in the global χ^2^. The diagnostic performance of this multiparametric approach was tested using the c-statistics for discrimination (area under the receiver operating characteristic curve; AUC).

Statistical analyses were performed using GraphPad Prism (version 8.3.0, GraphPad Software, Inc., San Diego, California, USA), SPSS (version 26, Statistical Package for the Social Sciences, International Business Machines, Inc., Armonk, New York, USA) and R package (version 3.5.3, R Foundation for Statistical Computing, Vienna, Austria).

## Results

### Baseline characteristics of the study participants

A total of 60 patients were recruited in the study between May 2017 and December 2019 (Additional file 1). All patients completed both ExCMR and invasive FFR assessment. The median time between ExCMR and FFR was 7 [6, 8] days. Of the 60 patients, 30 (50%) were FFR-positive: 14 (47%) one-vessel disease (1VD), 9 (30%) two-vessel disease (2VD) and 7 (23%) three-vessel disease (3VD). Patients had intermediate CAD pre-test probability based on the CAD Consortium Clinical Pre-test Probability Score (mean 38 ± 22%). Patients who were FFR-positive had higher pre-test probability compared to FFR-normal (45% versus 30%, *P* = 0.005). Non-ischemic LGE was present in more patients who were FFR-normal compared to FFR-positive (17 versus 7% respectively; *P* < 0.001). Of note, native T1 and ECV values were similar between the groups (*P* > 0.05; **Table ****1**); and there was no significant difference in native T1 between ischaemic and non-ischaemic regions (1014 ± 39 ms versus 1021 ± 30 ms; *P* = 0.334).

**Table 1:** Clinical characteristics and CMR parameters at rest and stress

	All patients (n = 60)	FFR-normal (n = 30)	FFR-positive (n = 30)	*P* Value
Clinical characteristics				
Age, years	59 ± 8	57 ± 9	61 ± 6	0.095
Males, n (%)	43 (72)	20 (67)	23 (77)	0.399
BMI, kg/m^2^	26.1 ± 3.7	26.1 ± 4.1	26.0 ± 3.2	0.901
BSA, m^2^	1.77 ± 0.18	1.80 ± 0.20	1.75 ± 0.15	0.342
Diabetes mellitus, n (%)	28 (47)	14 (47)	14 (47)	1.000
Hypertension, n (%)	49 (82)	23 (77)	26 (87)	0.325
Dyslipidaemia, n (%)	50 (83)	25 (83)	25 (83)	1.000
Smoking, n (%)	17 (28)	8 (27)	9 (30)	0.774
Family history of CAD, n (%)	26 (43)	13 (43)	13 (43)	1.000
Pre-test CAD consortium clinical score, %	38 ± 22	30 ± 20	45 ± 22	0.005
Medication, n (%) ACE inhibitor ARB Statin Other lipid-lowering drug Beta blocker	7 (12) 22 (37) 53 (88) 11 (18) 41 (68)	6 (20) 9 (30) 25 (83) 3 (10) 19 (63)	1 (3) 13 (43) 28 (93) 8 (27) 22 (73)	0.044 0.284 0.228 0.095 0.405
Systolic blood pressure, mmHg (rest)	139 ± 16	141 ± 14	137 ± 18	0.394
Diastolic blood pressure, mmHg (rest)	79 ± 13	79 ± 13	79 ± 11	0.943
Heart rate, bpm (rest)	73 ± 12	74 ± 11	72 ± 12	0.473
Rate-pressure product, mmHg/min (rest)	10,106 ± 2141	10,377 ± 2004	9835 ± 2271	0.330
Systolic blood pressure, mmHg (stress)	174 ± 26	175 ± 29	172 ± 23	0.650
Diastolic blood pressure, mmHg (stress)	111 ± 29	115 ± 30	107 ± 28	0.319
Heart rate, bpm (stress)	122 ± 19	123 ± 19	121 ± 20	0.558
Age-predicted maximal heart rate, %	76 ± 11	76 ± 10	76 ± 12	0.953
Rate-pressure product, mmHg/min (stress)	21,300 ± 5017	21,721 ± 5324	20,879 ± 4743	0.520
CMR parameters at rest				
Indexed LV mass, g/m^2^	50 ± 12	50 ± 14	49 ± 9	0.765
Indexed LV EDV, mL/m^2^	76 ± 15	76 ± 15	76 ± 15	0.892
Indexed LV ESV, mL/m^2^	33 ± 12	31 ± 10	35 ± 13	0.201
Indexed LV SV, mL/m^2^	43 ± 8	44 ± 9	41 ± 8	0.102
LV EF, %	57 ± 9	59 ± 7	55 ± 9	0.039
Indexed RV EDV, mL/m^2^	77 ± 14	78 ± 17	75 ± 11	0.432
Indexed RV ESV, mL/m^2^	34 ± 10	34 ± 10	34 ± 9	0.906
Indexed RV SV, mL/m^2^	42 ± 8	44 ± 9	41 ± 8	0.110
RV EF, %	56 ± 8	57 ± 7	55 ± 9	0.228
Cardiac index, L/min/m^2^	3.1 ± 0.7	3.2 ± 0.7	3.0 ± 0.7	0.212
CMR tissue characteristics				
Late gadolinium enhancement Ischaemic, n (%) Non-ischaemic, n (%)	13 (22) 7 (12)	0 (0) 5 (17)	13 (43) 2(7)	< 0.001
Native T1, ms	1020 ± 28	1018 ± 28	1022 ± 29	0.594
Extracellular volume, %	27.9 ± 2.9	27.7 ± 2.7	28.1 ± 3.1	0.657
CMR parameters at stress				
Cardiac index, L/min/m^2^	6.2 ± 1.2	6.7 ± 1.3	5.8 ± 1.0	0.003
Peak_CI_, percentile	8 [3, 19]	18 [7, 22]	5 [2, 8]	< 0.001
Change in cardiac index, %	104 [72, 140]	110 [89, 141]	87 [64, 145]	0.255
Change in indexed SV, %	26 [16, 43]	29 [18, 42]	23 [14, 48]	0.706
Change in LVEF, %	21 [13, 36]	28 [16, 39]	19 [10, 36]	0.119
Perfusion defects, n (%)	18 (30)	2 (7)	16 (64)	< 0.001
Regional wall motion abnormalities, n (%)	30 (50)	3 (10)	27 (90)	< 0.001

*ACE* Angiotensin-converting-enzyme, *ARB* Angiotensin II receptor blockers, *BMI* Body mass index, *BSA* Body surface area, *CAD* coronary artery disease, *EDV* End-diastolic volume, *ESV* End-systolic volume, *FFR* Fractional flow reserve, *LV* Left ventricular, *LVEF* left ventricular ejection fraction, *Peak*_*CI*_ Peak exercise cardiac index percentile-rank, *RV* Right ventricular, *SV* Stroke volume

### Cardiac Response to Exercise CMR

The ExCMR scan took 62 [56, 70] mins, with the stress component completed within 10 [8, 13] mins. Exercise stress was prematurely terminated in 4 (7%) patients due to symptoms. Peak exercise heart rate (HR) achieved was 122 ± 19 bpm, corresponding to 76 ± 11% of the age-predicted maximal heart rate (APMHR). Peak rate-pressure product was 21,300 ± 5,020 mmHg/min. Patients in both groups achieved similar peak HR, peak blood pressure and peak rate-pressure product (Table 1). ExCMR protocol was well-tolerated by all patients (median satisfaction score of 4 [3, 4]). The agreement between readers was substantial for perfusion (κ = 0.68) and nearly perfect for RWMA (κ = 0.85) and LGE (κ = 1.00).

Of all the ExCMR quantitative parameters, only Peak_CI_ correlated positively with FFR (r = 0.401; P = 0.002). No FFR-positive patients had a Peak_CI_ above 35^th^ percentile specific for age and sex. Moreover, Peak_CI_ decreased significantly with more severe CAD (1VD: 8^th^ [3, 9] percentile; 2VD: 4^th^ [2, 10] percentile; 3VD: 2^nd^ [0, 3] percentile; P = 0.045). Perhaps not surprisingly, exercise stress-induced RWMA, perfusion defects and myocardial infarction on LGE were markedly more prevalent in FFR-positive patients (Table 1). Conversely, there was no correlation between FFR values and segments of perfusion defect (ρ = -0.015; P = 0.0937) or infarction on LGE (ρ = 0.07; P = 0.807). The other ExCMR measures (change in indexed LV stroke volume (SV), LVEF and cardiac index) did not differ significantly between those patients with FFR-positive and FFR-normal (Table 1).

### Diagnostic characteristics of multiparametric exercise stress CMR parameters

**Fig. 2:**
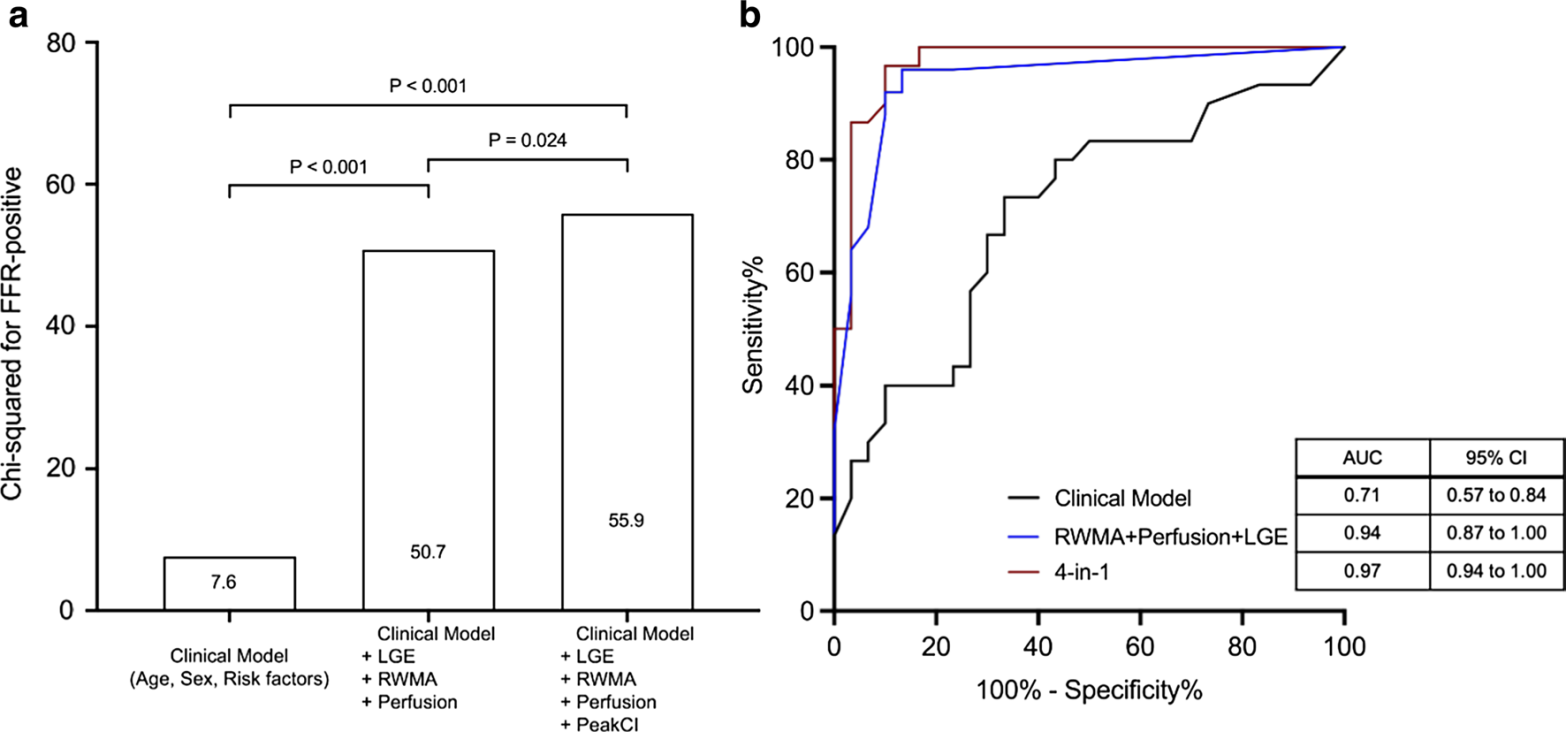
Test Performance of Exercise CMR. **a** Change in global χ^2^ value of the multiparametric “4-in-1” approach consisting of inducible regional wall motion abnormalities (RWMA), perfusion defects, late gadolinium enhancement (LGE) and peak exercise cardiac index (Peak_CI_). The clinical model consisted of age, sex and coronary risk factors (diabetes, hypertension, dyslipidaemia and smoking history).** b** Receiver operating characteristic curves to compare the discriminative abilities of the different models. A multiparametric model consisting of inducible RWMA, perfusion defects, LGE and Peak_CI_ offered the highest discrimination between those with and without significant coronary artery disease confirmed on fractional flow reserve

**Fig. 3:**
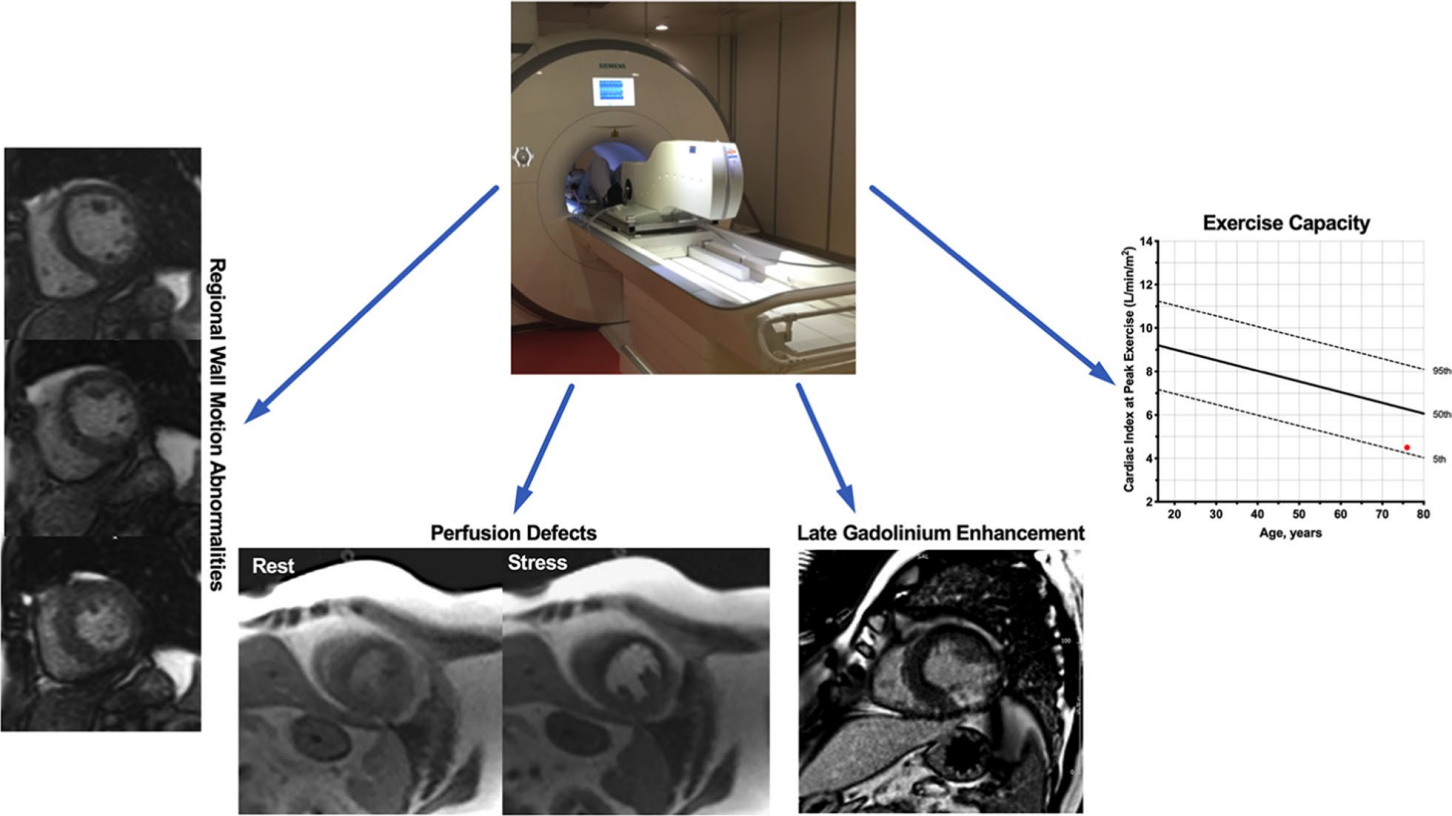
Central illustration. Multiparametric exercise CMR protocol. The multiparametric exercise cardiovascular magnetic resonance (ExCMR) protocol consists of regional wall motion abnormalities, exercise capacity, perfusion defects and late gadolinium enhanced patterns

Of all the CMR parameters assessed, only LGE, stress-inducible RWMA, perfusion defects and Peak_CI_ maintained an independent association on multivariate analysis with CAD defined by FFR (Table 2). Indeed, this multiparametric “4-in-1” approach (**Central Illustration**) demonstrated incremental diagnostic value over a clinical model. As a marker of exercise capacity, Peak_CI_ demonstrated incremental diagnostic value over inducible RWMA, perfusion defects and LGE (Fig. 2). The “4-in-1” model had the highest diagnostic accuracy [AUC 0.97 (95% CI: 0.94–1.00)], although the difference was non-significant when compared to the combination of conventional stress CMR parameters (inducible RWMA and perfusion) and LGE [AUC 0.94 (95% CI: 0.87–1.00); *P* = 0.136 for the difference between AUCs]. Similar results were observed when patients were stratified to above median APMHR [0.99 (95% CI: 0.96–1.00) and below median APMHR [0.95 (95% CI: 0.88–1.00); *P* = 0.339 for difference between AUCs] (Fig 3).

**Table 2:** Univariate and multivariate analysis of ExCMR parameters at rest and stress

	Univariate		Multivariate	
	OR [95% CI]	*P* value	OR [95% CI]	*P* value
LVEF	0.94 [0.88–1.00]	0.058	–	–
RWMA at rest	16.18 [0.69–378.80]	0.084	–	–
Late gadolinium enhancement	7.76 [1.98–30.42]	0.003	6.15 [0.74–51.37]	0.030
Peak_CI_	0.90 [0.83–0.96]	0.003	0.93 [0.84–1.03]	0.079
Change in cardiac index	1.00 [0.99–1.01]	0.355	–	–
Change in SVi	1.00 [0.98–1.02]	0.869	–	–
Change in LVEF	0.98 [0.95–1.01]	0.167	–	–
RWMA	61.74 [12.48–305.51]	< 0.001	22.90 [3.09–169.52]	0.010
Perfusion defects	34.19 [7.16–163.37]	< 0.001	6.77 [0.81–56.66]	0.020

*CI* Confidence Interval, *LVEF* Left ventricular ejection fraction, *OR* Odd ratio, *Peak*_*CI*_ Peak exercise cardiac index percentile-rank, *RWMA* Regional wall motion abnormalities, *SVi* Stroke volume index

## Discussion

EMPIRE is the first study to examine the potential of a multiparametric in-scanner ExCMR protocol to diagnose significant CAD, when compared with the gold standard invasive FFR. We have demonstrated that in patients with intermediate pre-test CAD risk, the ExCMR multiparametric approach consists of LGE patterns, stress-inducible RWMA, perfusion defects, and exercise capacity offered the highest incremental diagnostic value over a clinical model and excellent diagnostic accuracy [AUC 0.97 (95% CI: 0.94–1.00), *P* < 0.001].

Exercise is the most physiological stress technique. In recent years, we along with others have demonstrated the increasing potential of exercise CMR [13–15, 27, 28]. Assessing regional wall motion abnormalities and perfusion defects in a single stress imaging is not routinely performed with conventional adenosine stress techniques. The current study demonstrated the feasibility of the in-scanner supine cycling exercise CMR protocol to assess both regional wall motion abnormalities and perfusion defects. Using in-scanner cycling exercise, there is minimal delay in imaging at peak stress and acquisition at every exercise stage may increase the sensitivity to detect RWMA. With treadmill exercise CMR, there is a likelihood of reduced sensitivity because of the small delay in transferring the patients to the scanner [29, 30]. Instead of relying on one parameter, the novel ExCMR protocol combines different diagnostic strengths of regional wall motion abnormalities, perfusion defects, LGE and exercise capacity in a single stress modality to improve diagnostic confidence in detecting significant CAD. The promising results obtained from ExCMR protocol could potentially alter the paradigm of ischaemia evaluation. It is noteworthy to highlight that subsequent management (initial medical therapy or invasive strategy) will need to be tailored to the individual patient, particularly in the background of recent ISCHEMIA trial [31].

In our study, FFR-positive patients had reduced exercise capacity assessed using peak cardiac index, with a stepwise decrease in Peak_CI_ according to severity of disease. This observation is consistent with findings from studies in exercise radionuclide angiography and cardiopulmonary tests [32, 33]. Unlike peak LVEF and other exercise parameters, Peak_CI_ is the only stress measure that incorporates a physiologic parameter (HR) in response to exercise stress [14]. At low exercise levels, SV and HR increase linearly with increased work rate. At higher exercise levels, oxygen supply–demand imbalance in the myocardial regions supplied by the stenotic vessels cause regional dysfunction and consequently, decreased SV. As a physiological response, HR increases to compensate for the decreased SV in order to maintain adequate cardiac output [34]. In patients with advanced CAD, chronotropic incompetence impedes adequate HR response to further increase cardiac output [35]. Although temporal and spatial resolutions are lower in real-time cine images compared to traditional segmented-bSSFP images, we have demonstrated in a previous study that excellent agreement between volumetric quantification between the two image acquisitions can be achieved [14]. Accurate measurement of SV and cardiac index at stress is a strength unparallel in other non-invasive imaging modalities. However, other cardiac conditions such as microvascular disease or cardiomyopathies without epicardial obstructive CAD may be associated with similar poor exercise response [15, 36]. Thus, Peak_CI_ cannot be used as a single measure of significant CAD.

Previous studies have demonstrated diagnostic and prognostic value of exercise capacity in patients with CAD [33, 37, 38]. Our study supports these observations. Peak_CI_ offered a small but significant incremental value over stress CMR parameters (inducible RWMA and perfusion) and LGE in diagnosing significant CAD. The significance of this finding will need to be confirmed in larger cohorts of patients with CAD.

The maximum HR achieved with ExCMR is of topical interest. The recommended HR criteria of 85% APMHR to define adequate stress was based on treadmill exercise [39]. As the haemodynamic responses to the different stress modalities are different, we expect the maximum HR achieved with supine cycling to be lower than upright cycling and treadmill [40, 41]. In this study, the patients with suspected CAD achieved 76 ± 11% APMHR compared with 83 ± 3% and 78 ± 7% APMHR in healthy subjects and athletes, respectively [15]. Despite achieving HRs lower than the recommended 85% APMHR, we have demonstrated similar diagnostic performance in patients stratified by median APMHR. There are postulates that supine exercise increases myocardial oxygen demand and the increased LV filling pressure in the supine position may decrease coronary perfusion gradient during diastole, precipitating myocardial ischaemia [41, 42]. These findings may suggest APMHR is not the only consideration when assessing the adequacy of exercise stress CMR.

### Study limitations

Free-breathing exercise stress perfusion image acquisition at high HR remains challenging to achieve optimal spatial and temporal resolution due to breathing and cardiac motion. This would affect stress perfusion image quality and results in difficulty in the interpretation of images and lower interobserver agreement of perfusion assessment as compared to RWMA and LGE. Newer imaging sequences such as simultaneous multi-slice [43] or compressed sensing [44, 45] will likely provide better spatial coverage whilst shorten readout duration to minimize motion. Our ExCMR imaging protocol was designed and conducted on 1.5T scanner. 3T systems are now widely available and offer higher signal-to-noise and contrast-to-noise ratios that may have theoretical strengths of improved diagnostic accuracy for perfusion imaging. This warrants future validation. This is a single center experience in patients with intermediate pre-test CAD risk and relatively high CAD prevalence. Further larger, multi-center studies are needed to confirm our findings in lower risk cohorts. However, a single center is also essential at this point to ensure strict adherence to imaging and FFR protocols, crucial standards to establish for a first study.

## Conclusion

ExCMR using supine cycle ergometer demonstrates feasibility in assessing multiple CMR parameters that in combination, have high accuracy in diagnosing significant CAD as defined by invasive FFR. Future studies are now needed to validate these findings.

## Supplementary Information

**Additional file 1.** Online Supplementary.

## Data Availability

The datasets used and analysed during the current study are available from the corresponding author on reasonable request.

## Abbreviations

APMHR: Age-predicted maximal heart rate
AUC: Area under the curve
bSSFP: Balanced steady state free precession
CAD: Coronary artery disease
CI: Confidence Interval
CMR: Cardiovascular magnetic resonance
ECG: Electrocardiogram
ECV: Extracellular volume fraction
ESV: End-systolic volume
ExCMR: Exercise stress CMR
FFR: Fractional flow reserve
HR: Heart rate
LGE: Late gadolinium enhancement
LV: Left ventricle/left ventricular
LVEF: Left ventricular ejection fraction
MOLLI: Modified Look-Locker inversion recovery
Peak_CI_: Peak exercise cardiac index percentile-rank
PSIR: Phase sensitive inversion recovery
RWMA: Regional wall motion abnormalities
SV: Stroke volume
SVI: Stroke volume index
VO_2_ max: Maximal oxygen uptake
