# Supplementary material for: Multiparametric exercise stress cardiovascular magnetic resonance in the diagnosis of coronary artery disease: the EMPIRE trial
Source: J Cardiovasc Magn Reson. 2021 Mar 4;23:17. doi: 10.1186/s12968-021-00705-8 (PMC7931509; doi:10.1186/s12968-021-00705-8)
Supplement: Supplementary file 1 — Additional file 1. Online Supplementary. [file 12968_2021_705_MOESM1_ESM.pptx]

## Slide 1
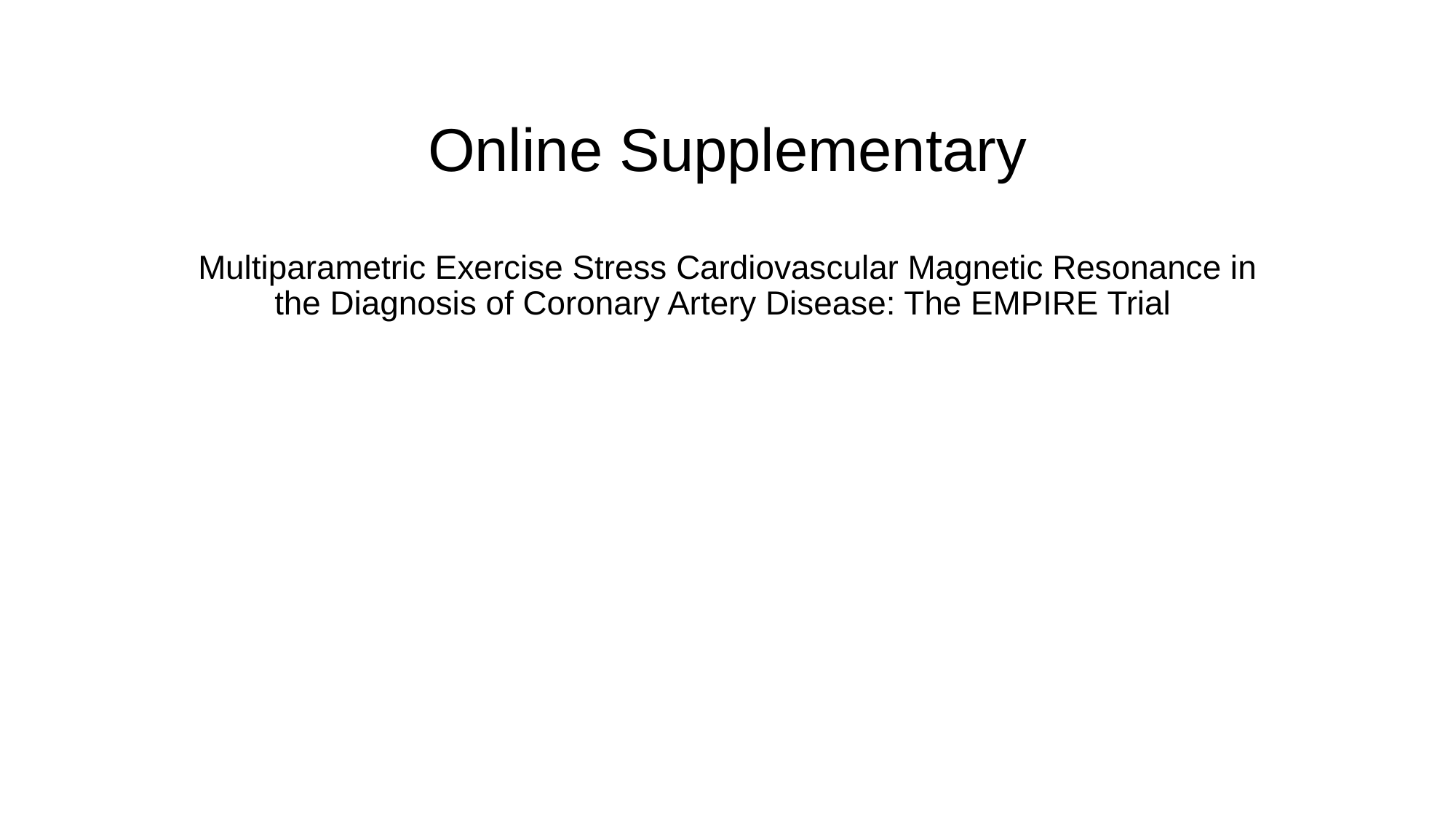

# Online Supplementary Multiparametric Exercise Stress Cardiovascular Magnetic Resonance in the Diagnosis of Coronary Artery Disease: The EMPIRE Trial

## Slide 2
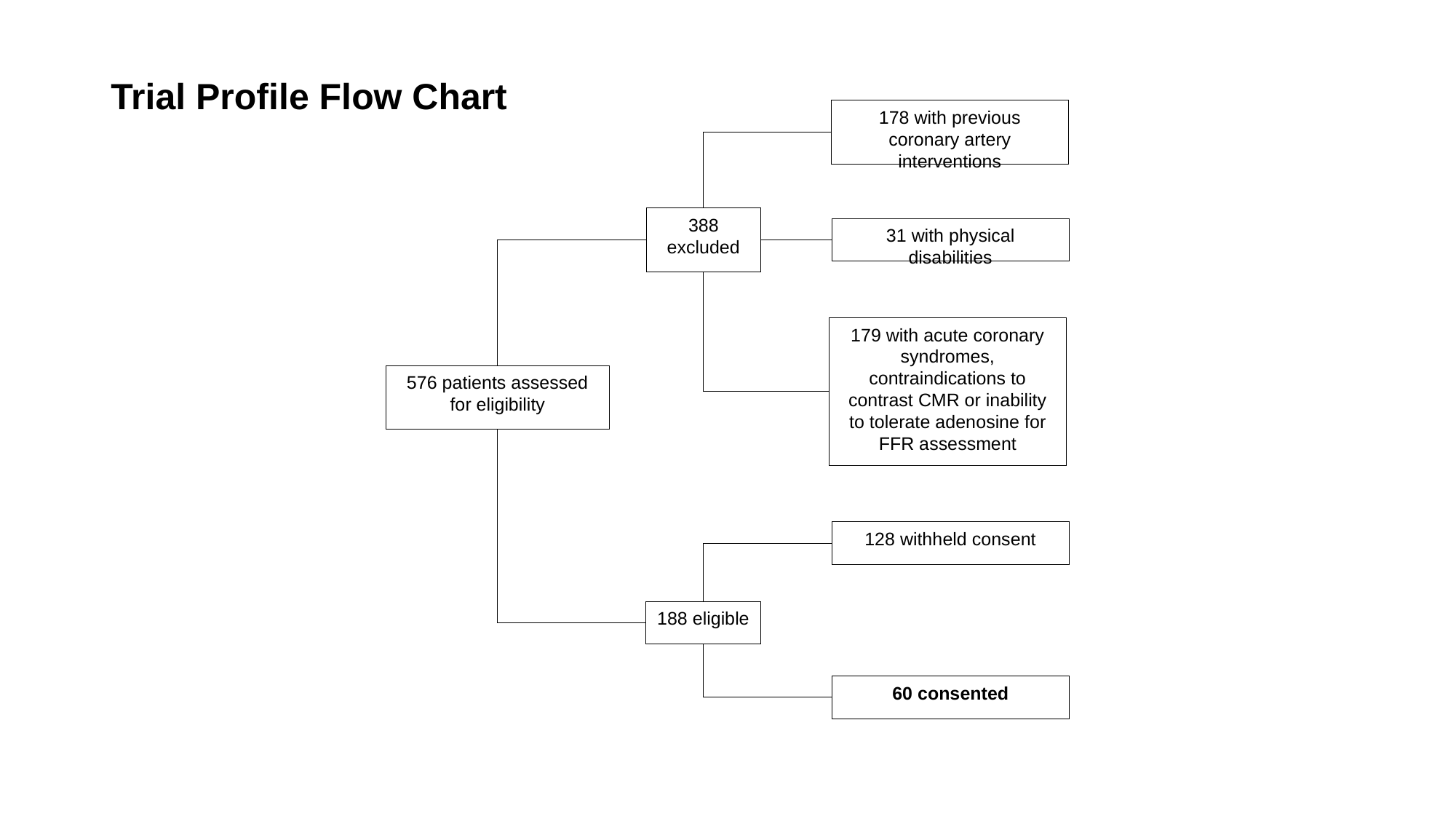

# Trial Profile Flow Chart
178 with previous coronary artery interventions
31 with physical disabilities
388 excluded
179 with acute coronary syndromes, contraindications to contrast CMR or inability to tolerate adenosine for FFR assessment
576 patients assessed for eligibility
128 withheld consent
188 eligible
60 consented

## Slide 3
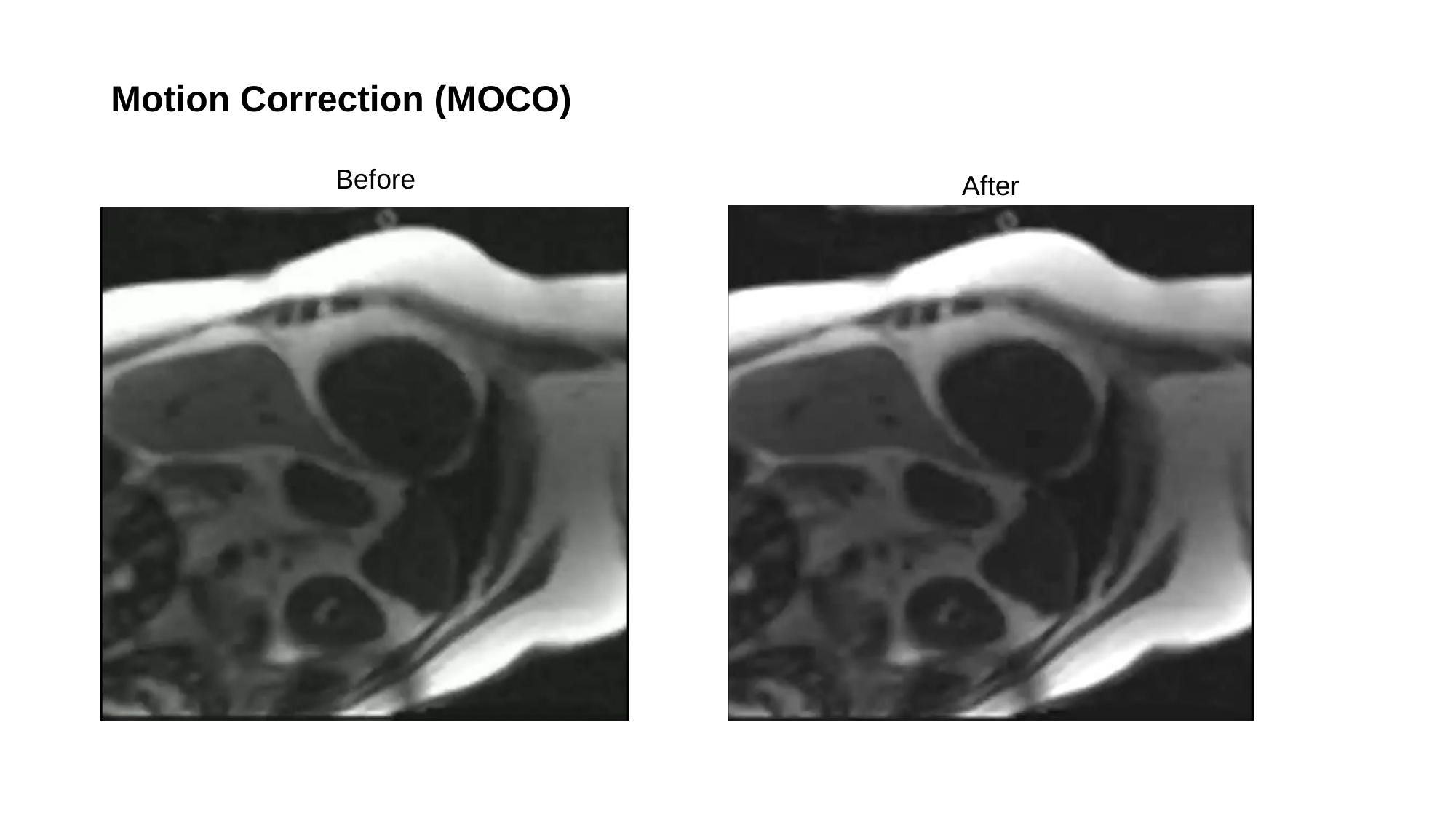

# Motion Correction (MOCO)
Before
After

## Slide 4
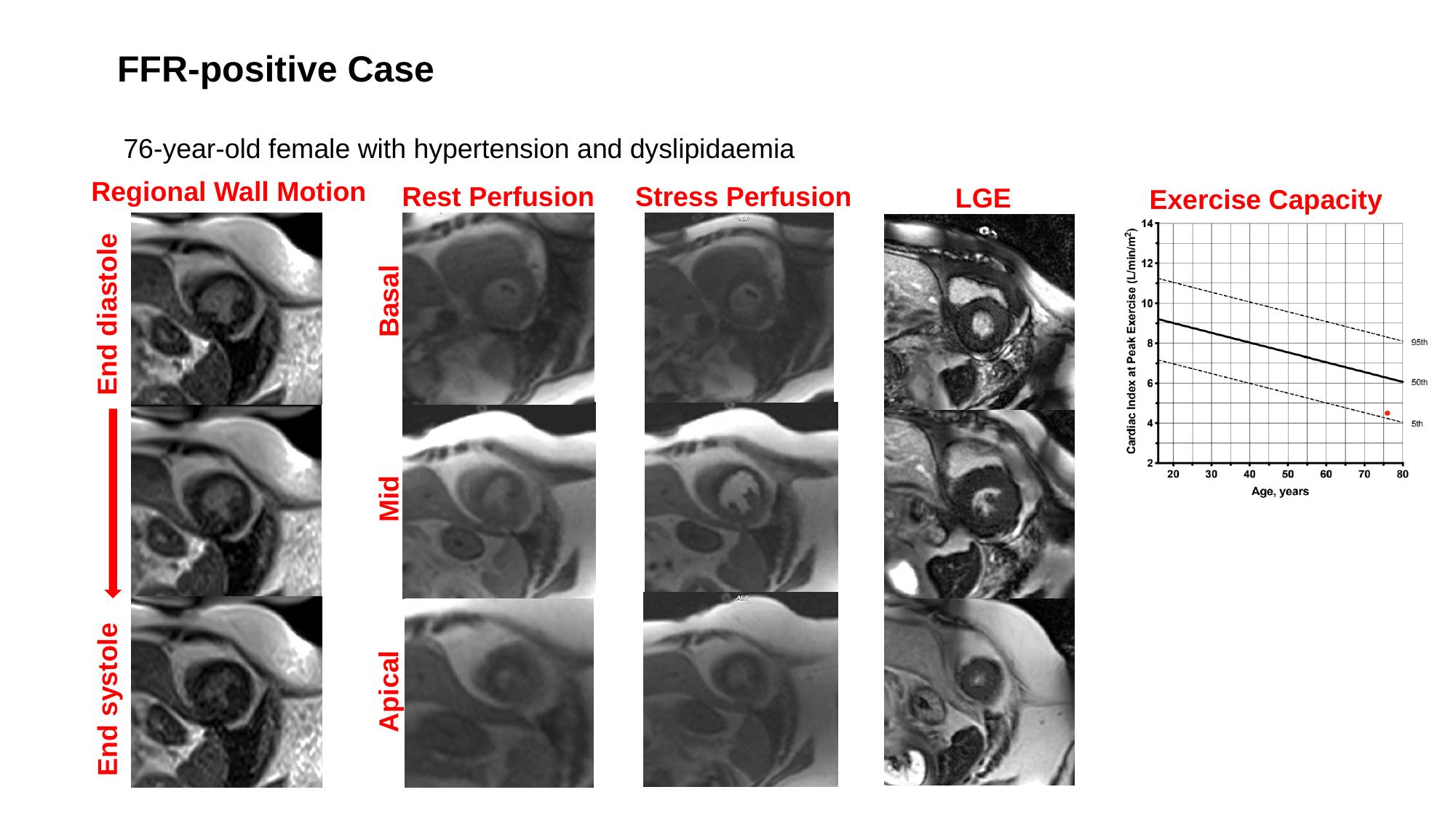

# FFR-positive Case
Regional Wall Motion
76-year-old female with hypertension and dyslipidaemia
Rest Perfusion
Stress Perfusion
LGE
Exercise Capacity
Basal
End diastole
Mid
Apical
End systole

## Slide 5
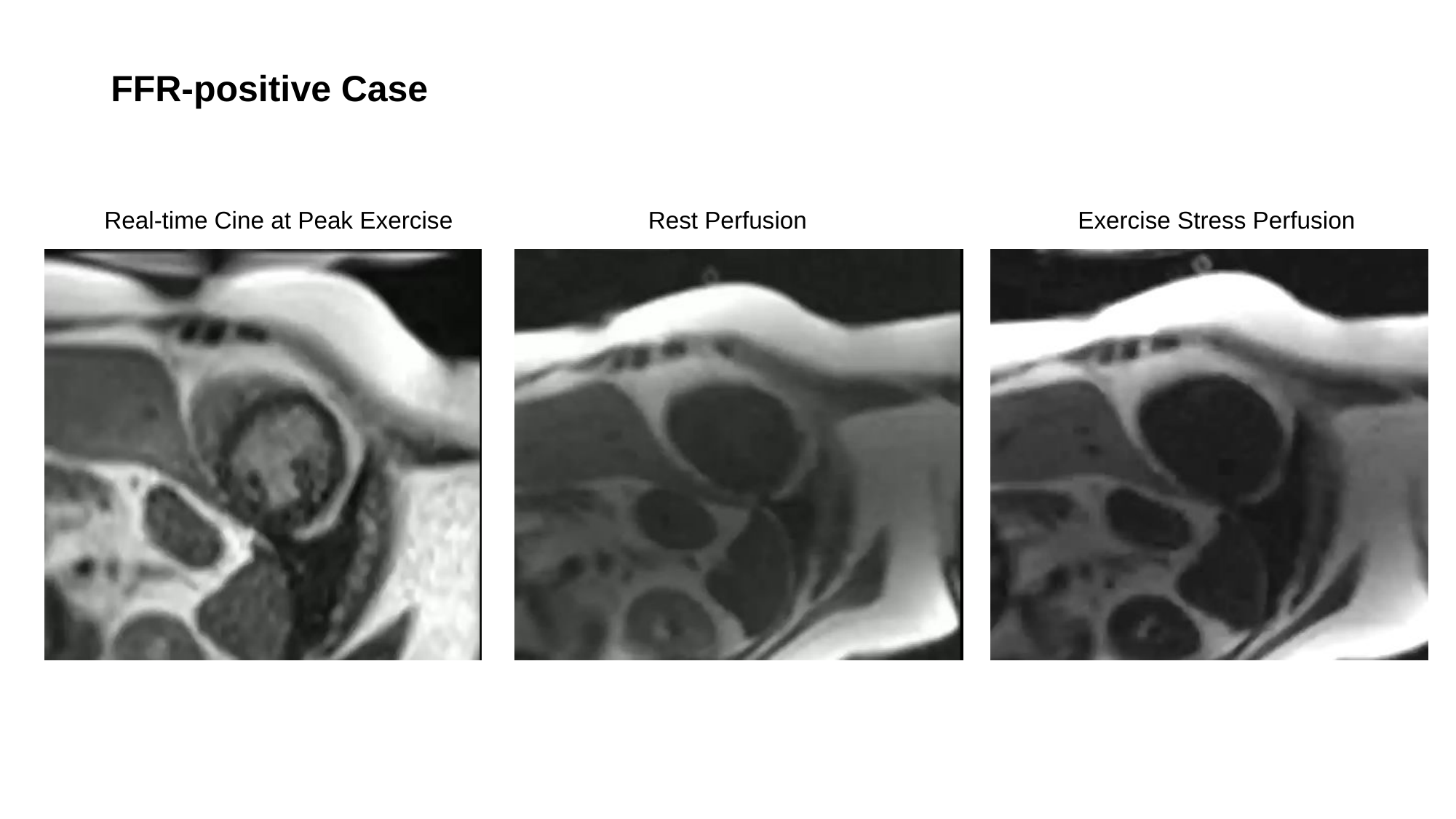

# FFR-positive Case
Real-time Cine at Peak Exercise
Rest Perfusion
Exercise Stress Perfusion

## Slide 6
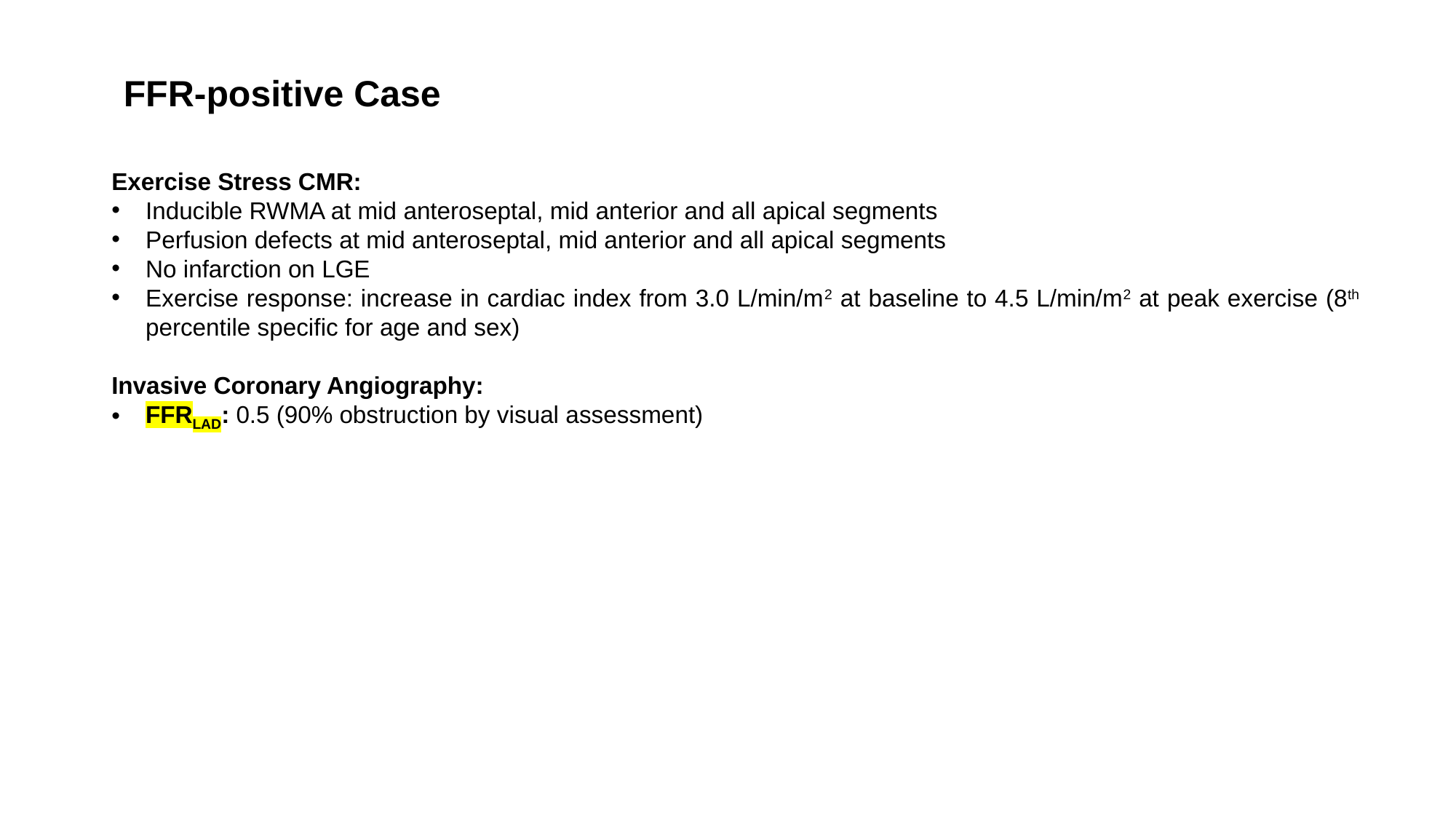

# FFR-positive Case
Exercise Stress CMR:
Inducible RWMA at mid anteroseptal, mid anterior and all apical segments
Perfusion defects at mid anteroseptal, mid anterior and all apical segments
No infarction on LGE
Exercise response: increase in cardiac index from 3.0 L/min/m2 at baseline to 4.5 L/min/m2 at peak exercise (8th percentile specific for age and sex)
Invasive Coronary Angiography:
FFRLAD: 0.5 (90% obstruction by visual assessment)

## Slide 7
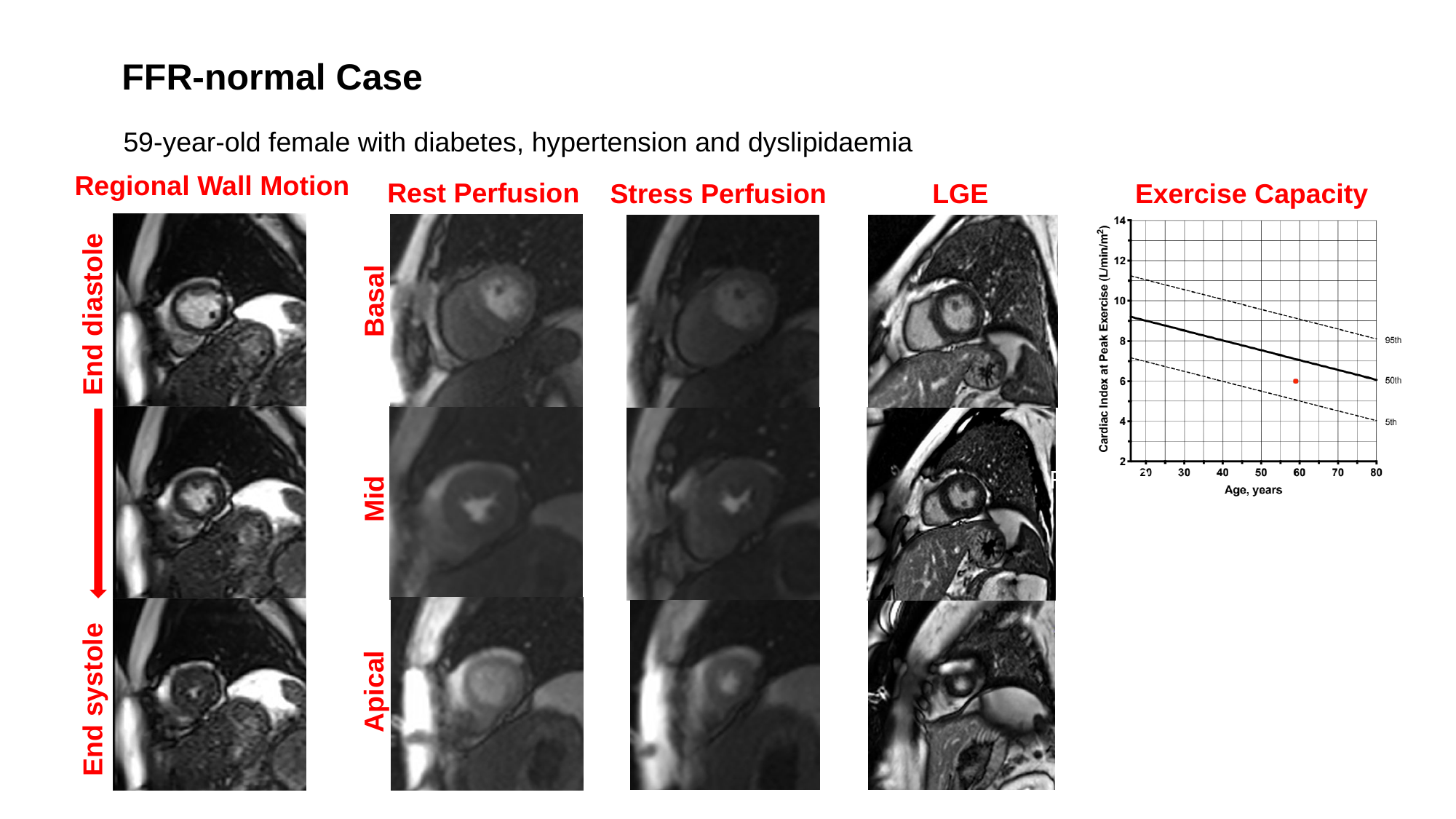

Regional Wall Motion
# FFR-normal Case
59-year-old female with diabetes, hypertension and dyslipidaemia
Rest Perfusion
LGE
Exercise Capacity
Stress Perfusion
Basal
End diastole
FFR = 0.86
Mid
Apical
End systole

## Slide 8
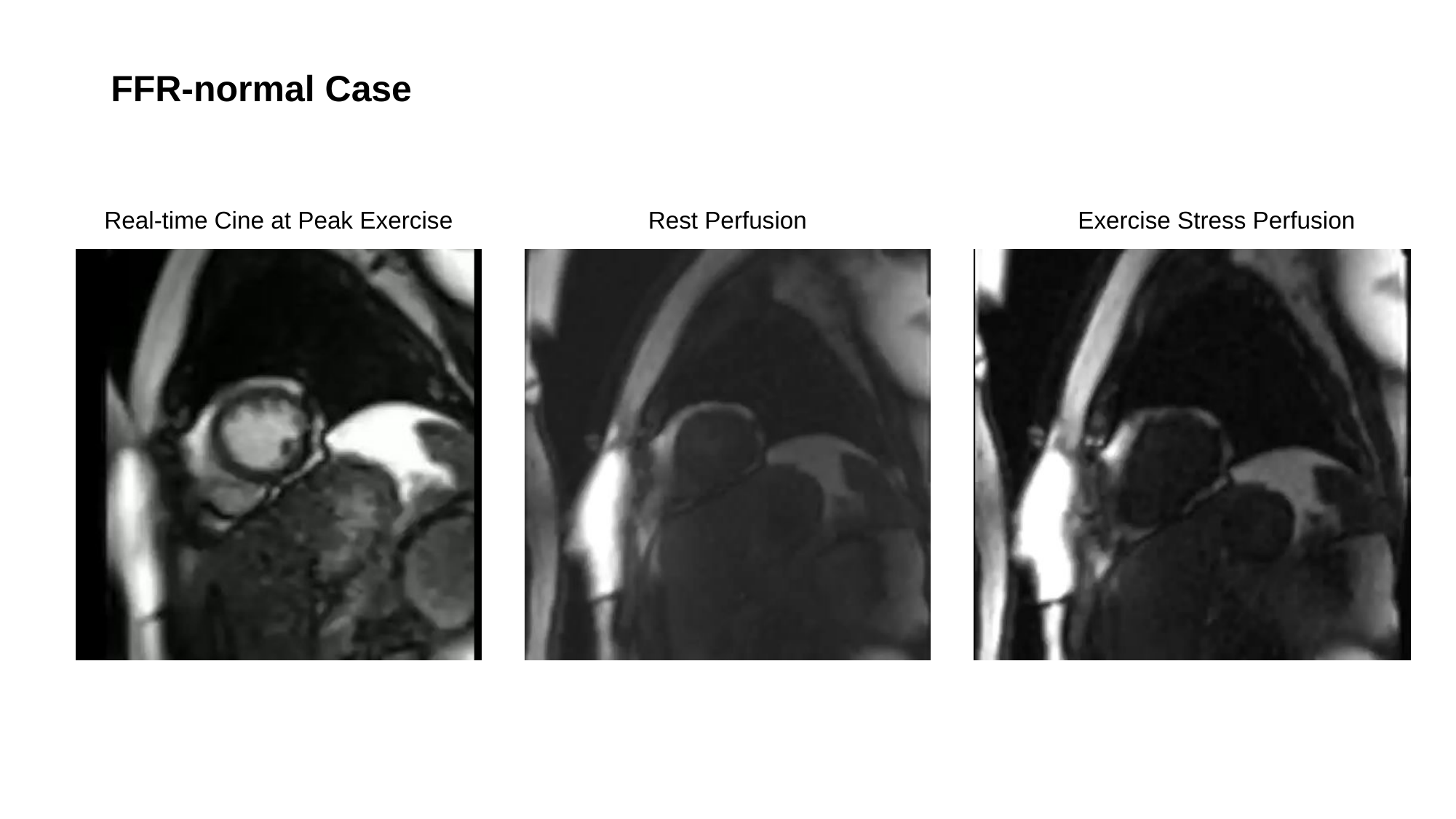

# FFR-normal Case
Real-time Cine at Peak Exercise
Rest Perfusion
Exercise Stress Perfusion

## Slide 9
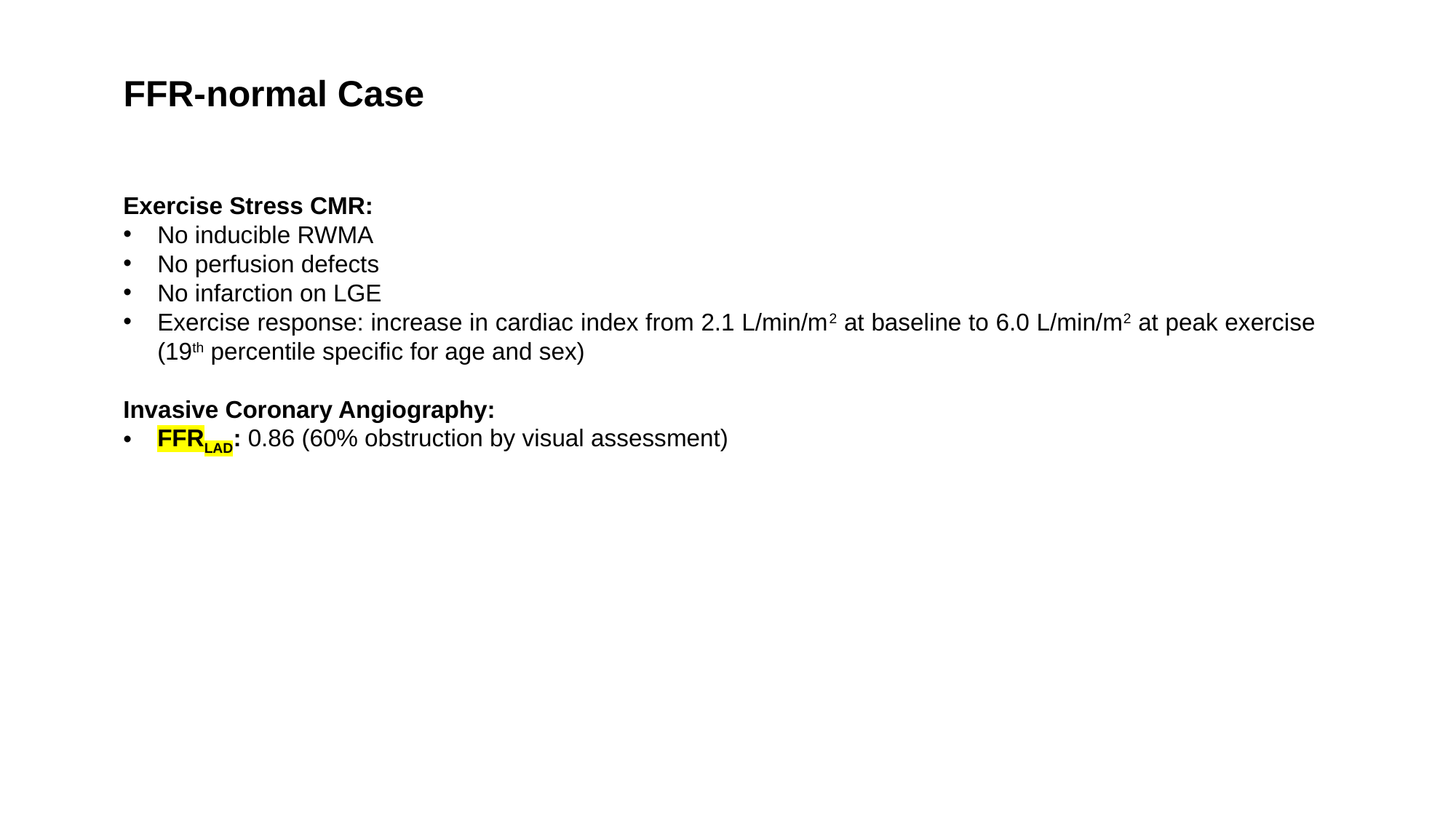

FFR-normal Case
Exercise Stress CMR:
No inducible RWMA
No perfusion defects
No infarction on LGE
Exercise response: increase in cardiac index from 2.1 L/min/m2 at baseline to 6.0 L/min/m2 at peak exercise (19th percentile specific for age and sex)
Invasive Coronary Angiography:
FFRLAD: 0.86 (60% obstruction by visual assessment)
